# Principles of Lithotrity

**Published:** 1832-01-01

**Authors:** 


PRINCIPLES OF LITHOTRITY; OR, A TREATISE ON THE ART OF EXTRACTING THE STONE WITHOUT INCISION. By *Baron Heurteloup*, Doctor of the Faculty of Medicine, Paris. Illustrated with Plates of the Instruments used in Lithotrity. Octavo, pp. 483. Five Plates. London, 1831.

THE present is a stirring century, an æra pregnant with great events. Men have learned and are learning to think for themselves; to appeal to reason and not fear her answers; to burst the bonds of civil and religious bigotry; and to laugh at childish reverence and pagan adoration for those dicta of their ancestors too often hallowed by the name of wisdom. Man, civilized man, is on the move. In the court, the camp, the city, and the grove, education is working a momentous change in the feelings, the pursuits, the manners, and the minds of those who are submitted to its kindly influence. Civilization is marching on; who can tell where is goal shall be? The political world is the scene of revolution, and, if coming events cast their shadows before, the changes to be will exceed in magnitude the changes that have been. But the overthrow of dynasties, and the alterations effected by reason or by force in the character of national or legal institutions, are equalled by those which we witness in the domains of science and art. In these we see indeed that the schoolmaster is abroad with his grammar and his birch; the first for the quick and the last for the dull. In the profession of the law the work of improvement, nay, of regeneration, is already begun; in the church, reformation is menaced, and perchance the secular arm may smite heavily the servants of the Apostles; in manufactures, in all those arts which, aided by philosophy and the more pure divisions of scientific knowledge, are made to contribute to the necessities, the comforts, and the luxuries of mankind: in these, we say, giant strides towards perfection have been made; in the higher departments of science itself the labourers are many and the harvest is not scant; in fine, we are surrounded by myriads of intellectual beings incessantly occupied in pushing the peaceful conquest of knowledge beyond the limits of her ancient reign—all breathes activity and mental energy: the press, the genius of modern civilization, is in the van; millions are in the ranks; the foes to improvement are few and flying.

In our own profession we are far from settled; we are not as we have been, we are not as we shall be. Our institutions are susceptible of improvement, and they will receive it. Each day brings some addition to our stock of knowledge, some fresh mine worked, some new ore found. Diseases are investigated after the inductive method, men of caution and of ingenuity are busy alike, competition is active, the results are beneficial. We need scarcely particularize what has been done for medicine and surgery in the latter part of the 18th and the 19th century, for they will tell their tale in the annals of our art.

Among the established improvements of our cotemporaries we must now begin to rank lithotrity. We are not of the party of those who cling to ancient prejudices, neither do we claim fellowship with the equally unphilosophic sect that embrace every new proposition without a careful and suspicious examination of its pretensions. Their's is a life of incessant disappointment; hopes blasted, prospects blighted, blot each page. When lithotrity was introduced to the notice of the profession, when the capital buzzed with its success in Paris, many were altogether incredulous, and many concluded that the Millennium for the stone was come. Years have passed away, professed lithotritists practise in France, and are established in London, and although the operation is not yet made parcel of the surgeon's education, nor become as it were the common law, yet it is not improbable that it soon may do so. It would yet be premature to pronounce confidently on its ultimate fortune, to declare that it would form the symbol of an important epoch, or pronounce that it would not survive its more immediate originators and advocates. We have not the gift of prophecy, or, if we have, we will not use it, but leave futurity to enlighten itself.

The present work of Baron Heurteloup is somewhat bulky, and we suspect that the Baron has not yet attained that art of condensation which pleases the public better than the printer. Five hundred pages on boring a stone, are well adapted for boring a reader. To say sooth, the Baron is a clumsy book-maker, and has yet to learn the taste of the day. The sages of the Row will smile when we tell them that the preface occupies forty-six pages. Nevertheless there are some observations in this preface not altogether unworthy of attention; and as they require little preface themselves we may introduce them to our readers.

"Whenever a new operation is brought to light, its existence is divided into two distinct periods. During the first of these periods general attention is only fixed on the efficacy of the means considered in an absolute manner, that is to say, independent of all distinctions of method in performing it. Is the operation successful or not? This is naturally the first question; and it is only secondarily that we inform ourselves in what proportion it succeeds. The second period, which is much longer, is that which elapses from the moment when facts have become sufficiently numerous to prove the efficacy, more or less certain, of a means of remedy, up to the period when other means, more perfect and of different origin, supply its place.

It is during this period that the art which has sprung to life from human invention, finds in that inventive faculty the means of improvement, by a continuation of the same process as that to which it owes its birth. It is when all the friends of science recover from the feeling of surprise with which they received the first idea, when they examine it more leisurely, regard it in its true aspect, and free it from all the clogs and impediments which private interests tend to throw in the way of its advancement.

Lithotrity has already passed through the first of these periods, it now begins to accomplish the second; but that it may be enriched, and become as rapidly perfect as it will allow, it is necessary that it should be freed from all the shackles of empiricism, and that the means of justly appreciating the value of each of the parts which may arise to forward the perfection of the process, should be infallible.

Lithotrity, truly considered in its essential character, and analysed, is but a purely mechanical affair, consisting only in the pulverization of a stone with more or less rapidity and gentleness towards the organ, which leads to the simple conclusion, that in order justly to appreciate the degree of perfection to which it is carried, the action of its agents should be examined independent of all collateral circumstances. This is what has not yet been done.

Hitherto it has been thought right to rest an opinion upon facts alone, without taking into consideration circumstances that might influence them. This, no doubt, was found sufficient, when proving the possibility of a cure by lithotrity was the only object; but now that it is necessary we should know the relative goodness of each of the instruments, more is required. To draw conclusions from the facts alone, is a vicious system, and must inevitably lead to error; for, besides that an author, whoever he may be, is always inclined by the weakness of human nature, to regard a fact which springs from himself, under a favourable aspect, very different in degree to that which he bestows on the property of another; a lithotritic fact especially has this peculiar inconvenience, that since it *takes place in the dark*, the operator himself may be deceived as to the nature of the observation he offers. The fact then, though simple, may thus undergo transformation, enlargement, fashioning, arranging, &c., till it appears to the eyes of surgeons a supernatural thing, and consequently nearly inimitable.

If, on the other hand, instead of consulting facts, such as they really are, we consider their number without taking note of the particular circumstances in which the practitioners were placed—such as the proportion of patients—the nature of the disease:—the amount of the number will prove a very illogical mode of reasoning with a view to the attainment of the truth.

It must be evident that too many circumstances may interfere to render a correct judgment possible by such means; that formed upon such grounds it must necessarily be false, even when the facts have been given with candour, truth, and especially with such accessory particulars as can alone give them value or render them current in science." *Preface,* xxxii.

Facts, then, true or false, (as false too many are,) are acknowledged by Baron Heurteloup as insufficient at present to determine the value of lithotrity, and more especially of the different methods of lithotrity. To remedy the deficiency of evidence, the Baron demands a kind of judicial combat, or, at least, a public demonstration, as he calls it, more resembling that ancient mode of deciding the right, than any other with which we are acquainted.

"By *demonstration*, I mean the application of lithotritic instruments on vesical calculi, and in the bladder of a cadaver, performed so that those who are appointed by the learned bodies to examine their efficacy, may see what passes in the interior of the organ, and thus appreciate in what degree of perfection the two principal conditions of lithotrity are fulfilled; that is to say, 1st, The absence of all lesion of the walls of the organ; 2dly, The rapid pulverization of the calculus.

I think that a commission chosen to examine and investigate all that relates to lithotrity, after having first compared and examined the means proposed by the different authors for placing the patient, and fixing the instrument while the pulverization is effected, may require them to shew, in the fullest detail, the manner in which they effect the crushing of the stone. They might first desire to see the pulverization effected on stones of different form and size, out of the bladder; and by afterwards placing calculi, varying in the same manner in their form and size in the bladder of a cadaver, they may examine the relative degree in which each of the authors, by means of their instruments, can acquire a preliminary knowledge of the stone or stones, as regards their shape and volume.*

They might then examine what influence the position of the patient has on the situation of the stone, and see which means of placing the patient is the best adapted to make these positions favourable to the success of the operation.

They can finally examine the instruments proposed by the authors, and require that they should make them act on small spherical stones—large and intermediate spherical calculi—small, middle-sized, and large oval stones—the same different sized flat ones—on a number of small round ones—and finally, on fragments.

To go through this examination, and to put the authors examined in the same situation as when they actually operate, they ought to be required to proceed to the destruction of the stones, without seeing any thing that is going on in the bladder, and without having received any previous information. The examiners placed so as to see and be able to touch the organ, which is fairly displayed, and distended with water, may judge by the bulges which the instrument makes in the posterior walls of the organ, whenever the instrument comes in contact. At each question of the examiner, the surgeon who operates ought to explain what he is doing in the bladder at the moment in which he is questioned ; and, if necessary, he ought to permit the bladder to be opened, to verify the accuracy of his description.

The examiners might even make the authors operate 'dry ;' that is to say, without any water in the bladder, which, thus emptied and opened at its posterior part, would allow them to examine, at leisure, the degree of harmlessness of the instruments for the neck of the bladder, the resources which they afford the surgeon, and finally, the ability which each displays in the double action of seizing the stone and pulverizing it.†

It would be after a report, circumstantial and well authenticated, had been made, in this spirit of analysis, that the authors of the various means to render lithotrity practicable would be classed, according to their respective claims, and that this branch of surgery not only might increase in its resources, and improve in its results, but be fairly appreciated in its present state."—*Preface*, xxxvi.

Whether this chivalrous challenge will be accepted we know not, but here we must quit the preface, and say something of the objects and constitution of the work. The aim of the Baron is to describe the instruments which he employs and the method of using them—the examination of the urethra and bladder and the calculi found in them—the circumstances which render the operation of lithotrity simple, difficult, or inadmissible—and, lastly, to detail some cases in which lithotrity has been had recourse to. It cannot be supposed that we can analyse this work, or that, if analysis were possible, it would be useful. We must omit altogether the description of the instruments, referring the reader to the work itself and the plates by which it is accompanied. We shall content ourselves with taking as brief notice as may be of the mode of examining the urethra and bladder, the circumstances deserving of attention in choosing or performing the operation of lithotrity,

---

* "It will be understood how necessary this preliminary knowledge is to the lithotritic operator ; since the shape and volume of the calculus exercises a powerful influence in his choice of an instrument that shall rapidly destroy the stone."

† "As I address myself more especially to the learned bodies of France, I will describe, in my French edition, all the points in lithotrity which they ought more particularly to examine and dwell upon."

and shall glance at a few of the cases related at the conclusion of the volume. In adopting this plan, we shall serve our readers, the author, and ourselves.

In some introductory observations, which form a sort of second preface, M. Heurteloup reviews the history of lithotrity, and treats of things which could not be conveniently treated of elsewhere. Amongst others, he draws a parallel between lithotrity and lithotomy, which strikes us as exhibiting some of the colouring of a partisan.

"Lithotomy requires to be performed in a favourable season; lithotrity may be performed at all times, with equal chance of success: the former requires that the patient should remain in bed for a month or six weeks after the operation, the latter needs no confinement, and often allows the patient to pursue his usual avocations. Lithotomy requires the patient to be kept on a rigid diet, while he who undergoes lithotrity is only moderately restricted; the recovery is often in the former case very tedious, whilst in the latter it is effected at once. Lithotomy often produces serious accidents, such as impotence, incontinence of urine, and urinary fistulæ; lithotrity, from its nature, cannot cause any of these consequences: the former, whatever may be the means employed, always requires a large and deep incision; the latter requires none. Lastly, lithotomy may cause almost instant death from hemorrhage; lithotrity cannot, under any circumstances, produce such a termination. We might carry this parallel still further, but the remarks we have just made, will, we think, be sufficiently convincing.

Thus the comparative advantages of the two operations cannot be balanced, and do not leave any doubt as to which the patient and the surgeon ought to choose." 4.

But may lithotrity be performed at all times, and with *equal success*, in health and in sickness—in sound bladders and diseased—with healthy kidneys and unhealthy ones? Has Baron Heurteloup, and have other lithotritists, *never* refused to operate on account of unpropitious circumstances, or would they never do so? We put these questions; let others answer them. Does lithotrity need *no* confinement, and is the cure effected at once? In short, is lithotrity so certain, so speedy, and so safe, as an ingenuous reader would be led, from the foregoing quotation, to consider it? Again we put a question; the answer is not with us.

It has been objected to lithotrity, that the instrument might break in the organ, or that fragments might remain, and become the nuclei of new formations. To the former, the Baron replies that no instrument has ever yet been broken. We may be mistaken, but we think that we have heard of two cases of this kind; in one, we "believe that the instrument was broken"—in the other, bent. The occurrence, then, is possible; but still the chance, with care and caution, must be inconsiderable. To the second objection M. Heurteloup replies, that facts hitherto have not been in its favour, and that if fragments did in some instances remain, they would merely require another application of the instrument. Perhaps the truth is, that the opponents of lithotrity dwell rather too much on these objections—the Baron rather too little.

The historical notice of the invention of lithotrity is rather instructive than curious. Arts, or even processes of any consequence or import to mankind, have always been perfected by slow degrees. To this there are no exceptions. The most brilliant theories are generally of comparatively gradual growth; the seed must be sown, and the soil must be apt. The knowledge of a fact is often diffused amidst many, before the man that gives it to the public connects his name with its origin, and comes to be considered as its sole inventor.

"In 1519, Alsaharavius expresses the idea only, of breaking small friable stones in the bladder with an instrument, (instrumentum subtile) of which, however, he gives us no description. In 1626, Sanctorious gives the plan of an instrument composed of a tube, one extremity of which divides into three flexible branches.* He says that if a fragment of a stone, or an entire calculus, descended from the kidneys, and was not expelled with the urine soon after its descent into the bladder, it might be extracted by first filling this organ with water, and then introducing the instrument he speaks of through the urethra, the branches of which, when arrived in the bladder, should be expanded, and applied close to the neck; he thinks that by then letting the water escape, the stone would be carried forward towards the three branches, seized by them, and extracted by withdrawing the instrument from the bladder. This author proposed also, and it is Haller that relates it, that if the stone should be too large to be extracted, it should be perforated with a stylet."† 12.

After this, successive improvements were proposed by various surgeons, but the modern instrument lays claim to two parents, M. Leroy d'Etiolles, and M. Civiale. Between these gentlemen there appears to be a difference, and we are not surprised that it is so. M. Heurteloup takes part with M. Leroy, and M. Civiale is designated as little better than a quack. We have not the means of deciding the question; if we had, we should want the time and the inclination. Yet we do not consider such disputes as frivolous, for reputation and fame are as dear as existence to the man of science.

When lithotrity was first bruited on English ground we heard only of M. Civiale's instrument. It consists essentially of branches to seize the calculus, with a peculiar drill worked by a drill-bow to perforate it. We doubted if this could be sufficient to *remove* a stone, to leave no fragment behind. We were told that it was. Let us hear what M. Heurteloup, himself no mean lithotritist, has to say.

"*To seize* a stone, to make *a hole in it,* to *drop it,* to *search* for and *retake it* in the bladder, in order to make another *hole* in it, to repeat this operation until it is broken, and afterwards to submit *each* of these broken portions to the perforation or pressure of the instrument, in order to render them small enough to be expelled—all this certainly appears a slow process, and one often painful in its application.

An instrument capable only of making a simple hole in the stone, appeared to me one of the smallest results that might be obtained by mechanical ingenu-

---

* "All these primitive instruments are drawn in the plates of M. Leroy's interesting work, entitled, 'Exposé des divers procédés employés jusqu'à ce jour pour guérir de la pierre sans avoir recours à l'opération de la taille (Paris, 1825).' This work may be procured at Baillière's, 219, Regent Street.

The instrument of Sanctorius is drawn (Pl. I., figures 16, 17, and 18.)"

† "Haller relates this of Sanctorius, but we do not find in his work any idea of the kind; Haller thought he had seen it in Sanctorius, because he mistook the figure, and took the stylet, which Sanctorius destines to reunite his triple branches, for a perforator. It results, therefore, that Haller, without being aware of it, invented lithotrity, whilst at the same time he believed it impracticable, for he adds—Speculationem puto meram."

ity, founded on the possibility of introducing through the urethra into the bladder a tube of three or four lines in diameter. This was, in my opinion, only the first step of science in a new path.

It struck me that the 'perce-pierre' was only sufficient when the stone did not exceed, or only slightly exceeded the diameter of the central drill, which was to perforate it, but that it became insufficient in proportion to the diameter of the calculus, which, as it became larger, would require a more frequent repetition of the perforations.

I apprehended, and many experiments afterwards proved it to me, that this instrument, besides its inefficiency, was not devoid of danger in its application; such for instance as fixing the hooked extremity of one of the branches in one of the holes made in the stone, and that also of engaging one of these hooks in the sinuses existing in some bladders. I was also convinced that the action of this instrument was often very deceitful, from the perforator falling into one of the holes made in a previous attempt. I saw that when the fragments were to be destroyed it too frequently happened that the portion was only grasped between two branches, so that it could scarcely be touched by the drill, which only acts in the centre." 33.

Of course we do not consider ourselves competent to offer a critical opinion on the subject, yet we venture to say, from no slight observation of books, of manners, and of men, that rival lithotritists will find imperfections in M. Heurteloup's contrivances, as well as in M. Civiale's. The following are the principles that have regulated the Baron in his improvements on or additions to the original instruments. We see in the mental process that guides the hand, no deficiency of philosophic connexion.

"I considered that if instead of breaking the stone by repeated perforations, I could devise some means of gradually and progressively enlarging a hole previously made, I should succeed in destroying the calculus at once, and by only seizing it once in the bladder; the centre being thus excavated and reduced to powder, the rest of the stone would fall in the form of a shell.*

I considered also that if after having reduced the stone to this shell, I could invent an instrument capable of crushing these flat and concave fragments with facility, as soon as they were taken, and without much time being spent in manœuvring, I should then have completed a system of destroying vesical calculi, which approached as near as possible to perfection; for on the one side I should have found the most rapid means of destroying whole stones and reducing them to fragments, and on the other, of again reducing these fragments sufficiently, for them to be easily expelled through the urethra.

I considered also, that if instead of operating with the patient on a common bed, on which he is always inconveniently situated, both for himself and the surgeon, I placed him on a small bed, expressly constructed in such a manner that he might lie at perfect ease, and if at the same time I was able to alter the position of the pelvis at will, I thought I should proceed more conveniently with the destruction of the stone, and should add to the chances of success all the advantages resulting from a position convenient both to the patient and to the surgeon; and lastly, I considered that if I could contrive some method of holding the instrument during its action upon the stone, more firmly than by the

* "The original idea of excavating the stone is, as we before stated, due to M. Leroy (D'Etiolle), but the elastic files he intended for this purpose, act very inefficiently on the calculus. The primitive idea might have been entertained by many persons; the difficulty, which I have been able to surmount, was to put this idea into execution."

*chevalet* proposed by M. Leroy, I should add still more to the improvement of this art, as without a firmer support than the *chevalet*, held in the hand of an assistant, my system of excavating the stone was impracticable.

I have by degrees achieved all these objects, and with sufficient success to have obtained several rewards from the 'Institut de France,' and particularly the great prize of surgery for the year 1828.

Thus in the first place I have substituted for the 'perce-pierre' which only destroys calculi of a certain size, by making *repeated* perforations in them, and which requires *frequent* attempts and *much* searching in the bladder, a system of excavating, which only requires the stone, especially when nearly spherical, to be once seized, in order to break it into pieces.*

Secondly, I have advantageously substituted for the same instrument, another peculiarly well adapted for destroying flat or concave fragments resulting from the action of the excavating instruments, and flat stones, which from their shape are extremely unfavourable for the 'perce-pierre.'

Thirdly, I have advantageously substituted for the inconvenient position of the patient on a common bed, a position which may be altered and adapted at will to the comfort of the patient, and the convenience of the surgeon, and *cæteris paribus* renders the operation more expeditious and more gentle.

Fourthly and lastly, I have advantageously substituted for the unsteady support of an assistant to maintain the instrument during the operation, a firm and immoveable 'point d'appui,' (fixed point,) useful when operating with the 'perce-pierre,' but indispensably necessary when operating with my excavating instruments.

Such is a most concise sketch of the four principal objects I have had in view. These, added to some new ideas with regard to the accessory treatment, such for instance as ascertaining the form of the bladder and stone before commencing the operation, that of injecting, that of producing the expulsion of the fragments from inert bladders, or the extracting them when lodged in the urethra, the means of discovering small stones or fragments in the bladder by a different method from that generally pursued, complete all the lithotritic apparatus of which I am the inventor. The means by which I have been enabled to accomplish all these objects will be explained in the course of this work." 39.

### On the Urethra and Bladder.

The Baron considers these parts in relation to lithotrity. As he makes some observations which are important to surgeons upon other accounts, we shall pause awhile, and extract what may be useful. A perfect acquaintance with the relative anatomy of the urethra is indispensably necessary to every practical surgeon. We are sorry to say that we witness daily proofs of the lamentable consequences of ignorance, or, at all events, imperfect information.

It was for some time imagined that a straight instrument could not be passed without difficulty into the bladder. This illusion has been effectually dispelled, yet still the quantum of difficulty is exaggerated. It is frequently imagined that the narrowest part of the urethra is the membranous portion. In point of fact, the least extensible is the anterior, and if a sound will pass through this, it ought to pass readily through the remainder of the canal.

---

* "The 'trois branches à virgule' is used for spherical calculi of from 8 to 12 lines in diameter; and the apparatus 'évideur à forceps' for those of from 12 to 24."

But the orifice of the meatus urinarius is frequently so small from natural conformation as to oppose the introduction of instruments of any size.

" Two anatomical dispositions contribute to contract the orifice of the urethra; first, we remark that this contraction may be caused by a fold of the mucous membrane, which unites the two lips of the meatus at its inferior angle, and according as this fold is large or small, so is the entrance of the canal: this is sometimes the only cause; but it occasionally happens that this membrane being divided, the orifice is no larger, and does not allow us to estimate the size of the urethra below the meatus. The additional obstacle is a kind of projection, formed by the two extremities of the erectile tissue which composes the glans penis; this, in some, projects considerably into the urethra; in others, though not so evident, still it impedes more or less the free introduction of the sound into the canal. It is on this projecting point that the sort of sensitive *sentinel* is placed, which warns the healthy person of the desire to void his urine, and the patient of the presence of a stone in his bladder, or of an inflammation of this organ; and it is this point which sympathizes in the affections of the other extremity of the canal.

The knowledge of this form of the meatus urinarius will afford us some useful precepts, which will be of service in those cases in which it is necessary to enlarge this opening." 49.

The Baron gives a very good description of the obstacles met with in the prostatic part of the urethra. It is highly deserving of attention.

" From the membranous portion of the urethra to the neck of the bladder the canal gradually increases in size: this is the part named by anatomists the prostatic portion; if we consider its absolute size, we should suppose that the instrument ought to pass it with ease after having traversed the membranous portion, but there are some circumstances which may prevent this, and which are in fact sometimes met with. This part of the urethra forms a cone, the larger opening of which is turned towards the bladder, and the smaller forms a continuation with the membranous portion. In the middle and lower part of this cone is that fleshy eminence named the *veru montanum;* but this is seldom sufficiently large to impede the progress of the sound, and besides it may be easily depressed when the third lobe of the prostate, which sometimes exists, is not enlarged.

A greater obstacle presents itself in that kind of *cul de sac* which terminates the prostatic portion nearest the bladder; this may impede the progress of the sound, and does so the more frequently in proportion to the small diameter of the instrument, and from there being two other additional causes which add to this difficulty.

There is often above the margin of the *cul de sac*, which forms the lower part of the neck of the bladder, a kind of fold of the mucous membrane, in the form of a crescent, and more or less developed in different individuals. This fold, which is a sort of valve, forms the posterior boundary of the *cul de sac,* and of course renders it deeper the more it is developed.

Besides this mechanical impediment which is still more augmented by the slight curve which the canal makes at this part, the introduction of the sound may be also impeded by another circumstance, namely, the contraction of the circular fibres of the neck of the bladder, and still more directly by the contraction of those muscles which give these organs the motions required for the due performance of their physiological functions. The levator ani muscle may, by the contraction of its anterior fibres, oppose the introduction of the straight sound, its action being to raise simultaneously the sphincter ani and the prostate; thus, when it contracts, it draws the gland up, and compresses the lower part of the canal against the upper, by which means it is contracted, not as in the membranous portion by a circular constriction, but, by being as it were in some degree flattened, which hinders the introduction of the sound.

Thus, after taking into consideration the obstacles which the internal structure of the urethra, and the laws which influence the contraction of its different portions offer to rectilinear catheterism, we see that, in the generality of cases, when the urethra is in a perfectly relaxed state, a straight sound of three or four lines in diameter ought to pass with facility." 52.

In order that a straight instrument may be passed into the bladder, the penis must be sufficiently depressed for the urethra to describe a right line from its orifice to the bladder. This is effected by depressing the urethra, where it is braced to the symphysis pubis by the suspensory ligament of the penis. Fortunately this is sufficiently extensible. When the instrument is in the bladder, that part of it embraced in the triangular ligament of the urethra is fixed or nearly so, consequently the instrument can only be made to turn as on a pivot, the extremities admitting of motion freely in contrary directions. If the straight sound when introduced is left to itself, it forms with the horizon an angle of from 18 to 20 degrees.

For the full and forcible expulsion of the urine the prostatic and membranous portions of the urethra must be fully dilated. In those who have stones or fragments in their bladders this dilatation is not fully effected, and this again becomes an obstacle to the ready escape of a small stone or fragment.

"Although it may happen that, when the urine is expelled freely, the fragments, if they are sufficiently minute to pass the neck of the bladder, are carried along by the stream of water, traverse the canal as through an inert tube, and are immediately cast out; yet if this expulsion is not sufficiently free, and the fragment, instead of getting into the stream when it commences to flow, only passes in just before the water stops, it may be impeded in some spot of the canal, which retains it more or less securely according to its degree of contraction, or according as the part is *absolutely* smaller. Generally a new flow of urine relieves the patient; sometimes, however, the canal contracts upon the fragment, and retains it, but dilates it sufficiently for the urine to pass.

The canal, like the neck of the bladder, possesses the *elective faculty* of retaining the fragments, and at the same time allowing the urine to flow. This *election* being a phenomenon dependant on sensibility, will lead us to lay down some useful rules to aid the expulsion of the fragments. Now, in considering the urethra with respect to its absolute capacity, that is, entirely devoid of contraction, the fragments traverse the canal, more or less rapidly, according as the part through which they pass is of greater or less diameter, or according as it is directed upwards or downwards; thus, they are frequently lodged at the entrance of the neck of the bladder, but seldom remain in the prostatic portion, which is large, and allows them to pass rapidly to the membranous portion, and the more so, as, when the patient is standing, the direction of this prostatic portion is from above downwards; they often lodge in the membranous, but quickly traverse the bulbous portion, which, although directed upwards, is the largest part of the urethra; lastly, they may be stopped according to their size, nearer or further from the meatus, for the urethra from the bulb to the meatus generally diminishes in size.

When the fragment is stopped in the canal, its progressive motion, towards the extremity of the passage, is produced either by the propulsion given to it by the stream of urine, or by that expulsive contraction which the whole of the canal possesses, and which belongs to its physiological structure; fortunate are those patients in whom it is well developed, and more especially those in whom there is only a slight degree of spasmodic contraction." 62.

The next subject investigated by M. Heurteloup, is the form and disposition of the bladder. The anatomical description of this viscus is worthy of attention, and the changes in form which it undergoes, under the varying quantities of its contents, are studied with ingenuity and described with care. We cannot do justice to this part of the work by any extracts. We shall content ourselves by giving a summary of the Baron's conclusions, referring to the work itself for the experiments and reasonings by which they are supported. 1mo. The fundus of the bladder is elevated during its simple retentive contraction. 2do. Whilst the desire to make water is moderate, which includes only a simple retentive contraction, the bladder diminishes in its antero-posterior diameter, at the expense of the anterior portion. 3tio. During a strong desire to pass the water, caused by the injection of too large a quantity of fluid, the fundus is contracted and elevated in its whole extent; and, during the violent contraction of the bladder, caused by the injection of a large quantity of fluid, the posterior part, particularly its upper portion, advances towards the neck. Hence it is seen that, during the violent contraction of the bladder, caused by the injection of a large quantity of water, the bladder, instead of being enlarged towards the inferior portion, diminishes in capacity; hence, also, when the bladder is in this state of contraction, if we let some of the water which it contains flow out, it increases in size at its fundus. This is in direct opposition to the generally-received opinion.

"It appears extraordinary that the bladder although distended with water, can, without any of this water escaping, contract to such a degree, that the sound can only be moved from the extent of from an inch to an inch and a half from the fore to the back part, and to about an equal extent laterally. As the water is incompressible, and the volume of the fluid in the organ remains the same, it must of necessity change its position during the state of contraction, and this is what in reality does take place; this water forced from the lower, flows towards the upper part of the bladder, or as I commonly express it, *the lower part of the bladder, by its contraction injects the upper.* Indeed, if we compare the muscular power of all that portion of the bladder, which is situated above a horizontal line drawn from the neck and passing round the viscus, to that which lies below this line, we shall readily conceive that when the organ contracts throughout its whole extent, one of these parts must yield to the other. In the upper part there is only the muscular coat, and even this is weaker at the apex than elsewhere; whilst in the lower portion there exists, not only the muscular coat, which is itself very much developed, but also powerful auxiliaries; it is not, therefore, surprising, that when these two halves of the organ are contending together, the weaker should yield to the stronger, and that that portion which presses least upon the liquid, should become distended notwithstanding its contraction.

If now we consider the great dilatation which may take place in the upper part of the bladder, as is proved by the large collection of fluid in some cases of retention of urine, we shall come to the important conclusion that *the bladder may receive a large quantity of fluid without being dilated at its lower part.*" 86.

We give the foregoing observation without comment, as we cannot pretend to determine whether M. Heurteloup is right in his statements or not. The following hint may be useful to lithotomists, as well as to lithotritists.

"We sometimes, at the moment of contraction, have the sensation of hard extended cords, on which the point of the sound strikes and rebounds, and which give to the hand a shock sometimes so forcible as to deceive us, and induce us to conclude that we have touched a stone. We know that these vibrating cords are nothing more than columns which exist in certain bladders. If, with the end of the sound, we examine the part where we experience this species of vibration, we find on each side of the fleshy pillar which produces it, a depression which is sometimes of considerable size, and into which the point of the sound passes; these depressions or cells are necessarily produced by the contraction of these *columnar bladders* upon the fluid they contain: the mucous membrane adhering closely to the kind of grating, which is formed by the fleshy bands composing its muscular coat, dips down where it is not supported, and projects where the muscular fibres are prominent. This, although a natural disposition, is one extremely important to be attended to in considering the operation of lithotrity; for when the muscular coat assumes this form in a very powerful manner, the simple depressions or cells become pouches in which the calculi may be lodged, and in which, moreover, the hooks of the forceps of an unskilful, careless, or unpractised operator may become entangled." 87.

From experiments on the dead body, he points out the positions assumed by stones of round and flat conformation. We cannot but believe that such observations, though useful and instructive, are not to be received with implicit confidence. We need scarcely insist on the differences that exist between a living bladder and a dead one. With respect to round stones, the Baron remarks:—First, that the more spherical a stone is, the more immediately it becomes placed before the neck of the bladder, and, consequently, being in the axis of the instrument, is more easily seized. Secondly, that the smaller a round stone is the easier it is seized, since its small size keeps it at a distance from the neck, and, consequently, it is at the spot where the branches are most expanded. The Baron also shews how far the position of stones may be purposely altered by the recto-curvilinear sound, and that round stones are more easily turned and more easily seized than flat ones. M. Heurteloup points out the advantages of elevating the pelvis, under certain circumstances, in order to facilitate the prehension of the stone. He lays down the following rules on this subject.

"First, *That it is extremely advantageous to raise the pelvis to an angle of forty-five degrees, in order to seize a round stone of an inch in diameter with gentleness to the organ, but that it is absolutely necessary to do so, in order to seize one of from eighteen to twenty-four lines.*

Secondly, *That this elevation is necessary, in order to seize flat stones, in direct ratio to their size.*

Thirdly, *That in order to seize the fragments of a stone with ease, this elevation is very serviceable for collecting them in that part of the organ most distant from the neck, and that it is often necessary to add to these means, shocks given to the pelvis, in order to become master of the most irregular of these fragments with facility.*

Fourthly, *To seize a stone less than an inch in diameter, the elevation of the pelvis is unnecessary, and may be disadvantageous, since in the horizontal position a stone of this size is naturally in relation with the two expanded branches contained in a straight tube.*

To give, in the last place, some further idea of the advantages of the elevation of the pelvis to 45 degrees, we may remark, that during this elevation the intestines no longer press their weight upon the upper and back part of the bladder; this induces us to employ these means in order to diminish or put a stop to the too violent contractions of the organ, since this position deprives, or at least lessens, the influence of one of the most powerful auxiliaries which add to its contraction." 110.

### ON CALCULI.

M. Heurteloup, after presenting a tolerably good account of the symptoms produced by calculi in the bladder, an account, by the way, which he published, with what we must consider bad taste, in the form of a little stitched pamphlet, proceeds to dedicate 32 pages to the examination of their physical properties. It must be obvious that the form of a calculus must be very important in a lithotritic point of view, nay, it must also be so to the lithotomist. But this is not all. Particular forms are more or less affected by calculi of particular chemical composition, and with that composition they are hard or soft, lamellated or confusedly crystallized. Calculi composed of lithic acid and of the phosphates when similar in shape, and of the size of from three to six or eight lines in diameter, do not produce the same effect on the organ.

"The smooth and hard uric acid gravel moves freely in the bladder, it falls into the neck, which it instantly blocks up during the expulsion of the urine; but being moveable and rolling about, it seldom remains in this position; it either bounds away, to return instantly, or its surface, which is most commonly polished, allows it, when placed in the funnel of the neck, to be forced out by the contraction of this part, which thus expels it by giving it a sliding movement; in this case, the irregularity which is caused in the stream of urine, is generally only instantaneous: for as it is often forced away, and again returns, the patient feels the attacks more frequently, but they are most commonly only of short duration. The uric acid gravel, which is dry, and of a density, but little greater than the fluid which surrounds it, rebounds about the neck, against which it strikes, either in the motions of the whole body, or during the expulsion of the urine; whence it is that the sensation of tickling or pinching at the end of the penis is generally felt by those patients who have red gravel of uric acid in their bladder, that this sensation is more severe, more sudden, and unexpected, and also that the stream of water is more frequently interrupted, but only for a short time.

Gravel composed of mixed phosphates is generally impregnated with the fluid which contains it; it is softer, and heavier, and is most commonly round; its surface is rough and uneven, and its physical properties, so different from those of uric acid gravel, considerably influence the symptoms which it produces.

Loaded with moisture, and consequently of great density, it moves less in the organ, it rolls more slowly; any shock given to the body is not so readily communicated to it as to a smooth lighter gravel; it alters its position with greater difficulty, and when it does alter it, it remains for a longer time in that particular situation in which any motion of the body may have placed it, or to which it has been forced by the action of making water.

As long as this species of gravel is not at the neck of the bladder, the patient suffers less; but, unfortunately, it is generally at this spot with which it is in more direct and permanent contact. Whilst the patient is walking, it falls heavily down upon this opening, and thus causes a longer continuance of the painful sensation, and this sensation is more dull and heavy.

But if the mixed phosphate gravel produces such distressing symptoms when influenced by the motions of the body, they become still more painful during the time of expelling the urine; and it is at this moment that the form and nature of the gravel can be ascertained by the symptoms which arise from its physical properties. Most commonly, the stream of urine is not stopped very frequently. If the form and specific weight of this gravel do not allow it to yield to the influence of the urine as readily as the uric acid gravel, and prevent it from passing so quickly to the neck, these physical properties cause it to remain, when once forced into that infundibulum which the neck forms during the act of expelling the urine; it does not readily escape from it; its chalky and uneven surface prevents the circular contraction of this part of the organ from forcing it out; it is locked in, as it were, between the parietes of the neck, and is held there the more firmly in proportion to the power of the contraction.

At first, the neck of the bladder does not feel its presence so acutely; it only surrounds and compresses it by its natural contraction, but this soon changes into a morbid action. The neck rebels against so disagreeable a companion; its sensibility increases, the membrane inflames, and then result spasms, a constant desire to make water, catarrh of the bladder, purulent urine, and, in a word, a collection of symptoms so severe, that one is quite astonished to meet with them in some patients who have only been afflicted with stone for a short time. We far less frequently meet with these symptoms when the bladder only contains uric acid gravel, which is rough; and we never observe them when the gravel is smooth and polished." 128.

Besides the differences thus occasioned others depend upon the round or flat form of the stone. The lithic acid gravel is generally flat, that of the mixed phosphates generally regularly or irregularly spherical. The symptoms produced by these gravels are different, and it is important to ascertain that difference, because it will serve to point out whether the stone is flat or spherical.

Round gravel of four or six lines readily passes to the neck of the bladder, and impedes the flow of urine the more, the more round and less polished the gravel is. Flat gravel of some size does not so readily pass to the neck, and when there, either produces no effect, or only very slightly impedes the stream. Sometimes it acts as a valve and renders the flow of urine intermitting; most commonly it lies lengthwise, and the stream is more or less twisted. By these symptoms the Baron asserts that we can conclude, à priori, and without sounding, whether the nucleus of the calculus has been round or flat.

Stones having acquired a certain size in the bladder are liable to modifications in form from several circumstances. The more moveable they are in the body the more they are disposed to be spherical, and vice versâ—when there are several stones they are worn away at several points of contact, but when of such a size as to be constantly in contact they are worn away at one spot; when many they are also generally polished, dense, and hard—lastly, the form is modified by the nature of the salt; oxalate of lime and the mixed phosphates tend to give a round form, the lithic acid, more particularly in the ratio of its purity, a flat one. When the bladder inflames and secretes much mucus the mixed phosphates are either deposited in distinct layers or in a shapeless mass. M. Heurteloup makes some remarks on the laws which regulate the formation of the phosphates. Having lately treated of this subject much more fully and satisfactorily than the Baron, in our analysis of Mr. Brodie's lectures, we need not advert to it further at present.

When the stone has attained some size it gives rise to nearly the same symptoms, whether it be round, oval, or flat. The bladder now contracts around the stone, and the latter is more or less fixed in consequence. If round, it can increase to a greater size before it is fixed; if flat it remains in the position in which it is most natural for it to lie, and its longest diameter is generally placed transversely. If oval or oblong it is usually in this position; if its greatest diameter should correspond to the antero-posterior diameter of the bladder, one of its ends sometimes projects into the neck whilst the other rests upon a part more distant from it, the calculus assuming the shape of a gourd. Inflammation of the bladder being excited by large stones, the mixed phosphates are secreted, and an irregular amorphous deposit thrown down upon the stone.

Round calculi are most readily seized and retained by a three-branched instrument. Flat stones are not so adapted, for four reasons: their position in the bladder—their form—their different diameters—and their hard and polished surfaces. A flat stone is more readily seized and held by forceps consisting of two branches. After some reflections shewing that round stones are calculated for being bored, flat ones for being crushed, the Baron concludes thus:—

"We have seen that round calculi can be most readily *seized* and *held* by forceps constructed with *three or four branches expanding, when projected from a straight tube;* and we have also seen that these calculi, from their form, and from the nature of the salts which compose them, allow of being hollowed from within outwards by the eccentric action of the instrument; now in this there is a fortunate coincidence, for these two mechanical means combine well together. In fact, the forceps thus constructed, holds the calculus in such a manner, that its axis, and that of the forceps, are in the same line, and any excavating instrument requires exactly, in order that its action may be complete, that these two axis should be the same.

We have seen that oval and flat calculi, on account of the two surfaces they presented, were seized with facility with an instrument consisting of two branches, and that these calculi yielded to the pressure of an instrument, but did so more readily to percussion. Here again is a fortunate coincidence, for the smaller the number of branches adapted to an instrument, the stronger they both are; and in order to destroy a stone by crushing, or by percussion, force is required, and the smallest number of branches we can employ is two.

Thus we see that the physical properties of calculi require the instruments to be differently constructed, in order to arrive at the important object of effecting their comminution in as short a time as possible, with the least movements possible, and in the most gentle manner for the bladder." 156.

The next portion of the volume is devoted to the analysis and description of the lithotritic instruments. These we cannot affect to describe; the curious must refer to the work itself. Here we may observe, once for all, that we cannot analyze these "Principles of Lithotrity;" they forbid it. We can only select such portions for extract, condensation, or comment, as appear to us to be likely to be instructive to professional readers, independent of merely lithotritic considerations. The following rules laid down by the author as necessary to be observed in the construction of instruments for this operation are deserving of insertion entire; indeed they cannot be curtailed. They may afford useful hints to such of our ingenious countrymen as may be devising alterations or improvements in the present apparatus.

"First Rule.—Rectitude in a sound, being: 1st, *the most favourable shape for bringing, without effort, the extremity of the instrument into contact with a great extent of the parietes of the bladder, and more especially with its fundus;* 2d, *for allowing the instrument to be moved backwards and forwards with facility;* 3d,

*for permitting it to be rotated on itself,* all instruments of lithotrity *must be straight in that portion of them which remains in the urethra during the operation,* for a curve would render them faulty in proportion as it impeded the execution of these three fundamental properties.

Second Rule.—Since the operation of lithotrity must be performed whilst the bladder is distended with water, it follows that the instrument must unite the necessary conditions for preventing the liquid, when once introduced, from escaping during the operation; hence, 1st, *it is necessary that the instrument should be, as nearly as possible, of the same diameter throughout, and that its vesical extremity should be as little enlarged as possible,* for the urethra through which an end much enlarged passed, would be but incompletely filled by the body of the instrument, and the water would consequently escape between the instrument and the sides of the canal; 2d, *the instrument must be of sufficient size to fill the canal, but not large enough to distend it, and render the movements difficult;* 3d, *it must finally be constructed so as to prevent the water from flowing out between its component parts.*

Third Rule.—Since urethræ vary in size, from two lines and a half, to four and a half, it follows *that the instruments must also present different diameters.*

Fourth Rule.—The contraction of the bladder being sometimes powerful enough to expel, between the canal and the instrument, almost all the water it contains, it is necessary *that every instrument of lithotrity should allow a fresh injection to be made into that organ, in order to distend it, so that the instrument may be closed without any danger.**

Fifth Rule.—Since the bladder varies in its antero-posterior diameter from two to three inches, on account of its contraction, *a lithotritic instrument must be constructed, so that its branches may expand sufficiently to seize the stone, and yet not project more than two inches and-a-half from the tube which contains them.* The instrument must also admit of *the projection of the branches being diminished, if circumstances should require it;* and this is the more requisite, since those bladders which contain the largest stones are generally the least capacious.

Sixth Rule.—When an instrument is expanded, and is in action in the interior of the bladder, the spot where the branches project from the tube is almost always in close contact with the neck of that organ; hence it is necessary *that the branches should have free motion, without affording a possibility of pinching at the part where they unite, and should always preserve the same distance between them, whether projected from, or drawn into the tube.*

Seventh Rule.—The bladder sometimes violently contracts during the operation of lithotrity, and presses upon the branches of the instrument which, when drawn towards the neck, are compressed by this part of the viscus, so that the surgeon is no longer master of the expansion of his forceps, and his manœuvres are totally impeded. It follows, therefore, *that the branches of every lithotritic instrument should be supported when they project from the tube and are expanded in the bladder, and that their expansion should not be entirely entrusted to the elasticity of the metal.*

Eighth Rule.—The object in constructing a lithotritic instrument being to bring a mechanical agent into action in the interior of the bladder, where the surgeon is unable to see in what manner it acts; it follows, *that every instrument for lithotrity must be graduated;* so that by means of certain signs placed at the extra-vesical extremity of the instrument, *the operator may be enabled to ascertain what is going forward at the intra-vesical portion.* Marks well contrived are therefore of the greatest importance.

---

* "I am here only alluding to the instruments which act by *progressive wasting* on the stone, and which consists of three or four branches, for these alone are apt to become entangled in the bladder."

Ninth Rule.—Some bladders are very irregular, and their superior and posterior parts, although distended with water, are sometimes forced from the sacrum towards the neck of the bladder, either by the contraction of the viscus itself, or by the pressure of the intestines which are pushed down by the abdominal muscles ; it follows, therefore, that an instrument, destined to seize large calculi, which require a large expansion of the branches, is faulty in its construction, *if the branch which is above terminates in a hooked extremity; it is the more faulty the further the branches project from the tube to grasp the stone, and the smaller the size of the bladder.*

Tenth Rule.—Since it is necessary, under certain circumstances, to discontinue the operation of lithotrity, either on account of a great degree of sensitiveness in the patient, or because of a violent and permanent contraction of the bladder, it follows, that it is not sufficient for an instrument to *grasp* the stone with facility, *but it must relax its hold with equal ease ;* and as this becomes more frequently necessary in operating on large calculi, this property must predominate more especially in those instruments destined to act in such cases.

Eleventh Rule.—Since the excavation of large calculi produces a very considerable quantity of powder, which would clog the action of the 'évideur' (excavator), it follows, that *an instrument intended to excavate such calculi, must afford the facility of injecting water into the bladder to wash away the superabundant powder, even whilst the instrument is in action.*

Twelfth Rule.—Since that portion of the instrument which destroys the stone by *progressive wearing away* must be removed as far as possible from the parietes of the bladder, it follows *that this action must proceed from the centre of the calculus to its circumference ;* for were it otherwise, the destroying agent would be in almost immediate contact with the interior of the organ.

Thirteenth Rule.—Since the action of the instruments upon the stone must be performed with the greatest regularity, both as regards the action of seizing and that of breaking, and since the exact performance of this must depend entirely upon the manœuvres of the surgeon, it follows that *no part of a lithotritic instrument must be left to act by means of springs, the movements of which are in a great measure independent of the surgeon's will.*

Fourteenth Rule.—Since it is necessary that the different parts of an instrument should play with facility one upon another, in order that the surgeon may have all the precision and delicacy of his tact, it follows that the instrument *must not only admit of this free and easy play,* but it must also be so constructed as to render it easy *to take it entirely to pieces, so that it may, in all its parts, be thoroughly cleaned.* Without the observance of this condition in an instrument, there cannot be sufficient precision in the manœuvres."* 168.

### ON THE CIRCUMSTANCES WHICH INTERFERE WITH, OR ARE FAVOURABLE TO LITHOTRITY.

Lithotrity is not equally applicable to all ages. It is generally, and for reasons which must partly be very obvious, inapplicable to children under

* " A great number of instruments have been invented to perform the operation of Lithotrity, and more especially in France, but they have proved unfit for use, as the greater part of them deviate from one or the other of the rules here laid down, and are thus the more defective and inapplicable, in proportion to the importance of the rule neglected; many of these instruments are faulty from the non-observance of 5, 6, 7, or 8 rules, and unfortunately operations attempted with them have sometimes caused the death of the patient; but can such fatal terminations be laid to the account of Lithotrity ?"

eight or ten years of age. The adult is the most favourable time of life for the operation, and the smaller the stone, and the earlier it is operated on the better. Old persons are less adapted for lithotrity on account of the deficient power of the bladder to expel its contents, and the fragments of the calculus.

Corpulency, which is an impediment to lithotomy, rather offers facilities for the performance of lithotrity; it it therefore no objection, at least in a healthy condition of the urinary system. A great degree of organic disease furnishes sufficient reason for shunning the operation, especially if several sittings are required. A strong calculous diathesis may also prohibit lithotrity, or give the surgeon pause before he ventures on it.

The state of the urethra requires consideration. If naturally of small size, the circumstance is unfavourable; a small orifice is inconvenient, and must be divided, and stricture of the urethra renders it difficult or even impossible.

"We have treated very few calculous patients by lithotrity who had, at the same time, considerable strictures; all such as we have treated were cases of small calculi, which could be easily destroyed; but although the canal was dilated to the extent of three lines and a quarter, the fragments were voided with considerable difficulty. They frequently lodged in the urethra, arising either from the strictured parts contracting on withdrawing the bougie, or from this part of the canal being without that kind of expulsive contractility which is observed in a healthy urethra, and which these patients possessed in a high degree. All the other patients had stones much too large, considering the state of the strictures, for lithotrity to be applicable.

It must now appear evident how difficult it is to decide upon the propriety of performing lithotrity in a patient with a stricture, before removing the stricture; since it is the calibre of the canal which influences the power of the instruments, and the facility with which the fragments will be voided. It is impossible to decide until the canal is dilated as much as it admits of. We must, therefore, in these cases of stone combined with stricture, when there is any doubt about lithotrity, first treat the stricture; and we may do this the more readily, as at any rate this treatment will be beneficial to the patient, even should we afterwards find that the operation is inadmissible. If, on the contrary, from the diseased state of the bladder, and the size of the stone, we at once decide that lithotrity ought not to be attempted, it will then be better to perform lithotomy at once, provided the stricture be not so complete as to prevent the introduction of the staff." 328.

A large scrotal hernia is an embarrassing, but not insurmountable obstacle.

"Stones lodged in the urethra give rise also to great, and sometimes even insurmountable difficulties, especially when the calculus has become imbedded in the canal, and can only be removed by an opening. We have met with a case of this kind. After the stone was removed, and the opening healed, this part of the canal was too much contracted to permit the operation of lithotrity to be performed, in order to remove the other calculi which were in the bladder. Lithotomy was resorted to in this patient. There is a very important consideration with regard to calculi thus lodged in the urethra, to which lithotomy itself gives rise. Before the art of pulverizing a stone in the bladder was introduced, the rule was, that, when a calculus became lodged in the urethra, great care was to be taken not to push it back again into the bladder, but to extract it by making an opening into the canal. This rule was given in order to avoid the operation of lithotomy, which must be performed if the stone be made to pass from the urethra into the bladder. But now that we are enabled, by more gentle means, to remove a stone, thus returned into the bladder, without subjecting the patient to the dangers of lithotomy, the rule ought to be precisely the contrary—that is, the stone ought, if possible, to be returned into the bladder, and no opening made to remove it; for, if this operation be trivial when compared with lithotomy, it is an important one when compared with lithotrity. The advantages of lithotrity are still more important when we consider that, when a stone, thus lodged in the urethra, has been removed by an opening, it is frequently found necessary to perform lithotomy besides, to remove other small calculi remaining in the bladder, the existence of which was not suspected, since the stone lodged in the urethra obstructed the passage, and prevented those in the bladder from being ascertained." 329.

A soft and fungous state of the mucous membrane of the urethra is disadvantageous for lithotrity; a very sensitive urethra is so also; but this extreme sensibility may generally be relieved or removed by the daily introduction of flexible bougies.

The state of the prostate has great influence on the performance of lithotrity. When enlarged it impedes the operation, and its various varieties of form and figure exert a material influence on the operation. "Not to be unjust, however," says the Baron, "towards the prostate gland, I will here terminate by remarking that it is too often accused of producing difficulties which result from totally different causes."

The condition and form of the bladder are, of course, of great importance. A large lateral diameter of the bladder is productive of great inconvenience and difficulty. Its form is sometimes injuriously altered by internal hæmorrhoids, or accumulations of fæces in the rectum. A bladder presenting pouches requires great care in the manœuvring of the instrument, and when it contains two or three cavities communicating with each other, it offers great difficulties both to the lithotritist and the lithotomist. A bladder of moderate size is the best adapted for the operation. A diseased state of the bladder deserves great attention. Chronic inflammation of the mucous membrane is common, and gives rise to considerations of importance.

"The first of these is, that a patient seldom has a stone and a catarrh of the bladder, without the latter being symptomatic of the former; it is only when the stone has existed for a long time, when the bladder is contracted and thickened, that the catarrh becomes idiopathic, that is to say, that it does not cease when the stone is removed. A proof of this is found in patients who have undergone the operation of lithotomy, and have been cured, but still retain a catarrh of the bladder after the wound has healed. These examples are rare, because, when the bladder is thus catarrhal, hypertrophied, and much diseased in its structure, it is seldom that patients recover after lithotomy. As long as the diseased state of the mucous membrane is not combined with a diseased state of the muscular and cellular tissues, producing the thickening of the coats of the bladder, and that rigidity which prevents its being distended, catarrh does not prohibit the operation.

We have, in fact, performed this operation on several patients who were thus affected, and who did not appear to have any increase of sensibility when the organ was touched; among these cases, two more particularly had so much catarrh of the bladder, that the mucus deposited each night at the bottom of the vessels weighed seven or eight ounces, these patients underwent the operation of lithotrity, and were relieved both from their stone and their catarrh.

In both, we observed, that each repetition of the operation was followed by an evident diminution in the quantity of mucus, it did not, however, entirely disappear till some days after the patients recovered. In these subjects it was as easy to distend the bladder as in other cases. We do not mean, by these examples, to prove that the bladder may be thus easily distended in every case of vesical catarrh, we only wish to shew, that this affection of the mucous membrane does not prohibit the use of the lithotritic instruments, when there is facility in distending the organ, for the disease is then confined to the mucous membrane only." 341.

When the urine is muco-purulent, with a disposition to hæmorrhage, the operation is prohibited.

"Thus, the mucus of the bladder only prohibits lithotrity, when the stone is very large, and when there is also contraction of the organ, arising from the thickening of its coats; it is quite clear, that if the stone be small, though the bladder be catarrhal and contracted, lithotrity is practicable, not only on account of the absolute smallness of the stone, but because, being small, it can only be of recent formation, and the contraction of the bladder is not caused by the thickening of its coats, but by the irritation arising from the presence of the calculus, which is then most commonly light, friable, and easily pulverized, but presents small crystals which resemble needle-points. When speaking of the different kinds of calculi, we explained the reason why stones of this sort caused so much irritation and catarrh of the bladder in so short a time." 342.

Paralysis of the bladder offering an obstacle to the discharge of fragments is very inconvenient. Vesical growths and polypi are occasionally met with, and require great care and prudence on the surgeon's part. Sometimes the mucous membrane of the bladder is in a soft fungous state, one very disadvantageous to lithotrity. A varicose state of the veins at the neck of the bladder is unfavourable, and various growths, or diseases around it are so likewise. In some patients the bladder is extremely contractile; this is obviously more or less inconvenient. Finally, the Baron winds up with a sort of summary of what the lithotritist should be, and what he may expect.

"His art exacts constant attention, continual care, great skill, and long study, to enable him to decide upon the propriety of undertaking the operation, or upon the propriety of continuing it when once begun. As long as the patient is in a favourable condition, middle aged, with a small stone, a large urethra, a moderate-sized bladder, well formed, and not irritable, the means of obtaining a cure may be easily chosen and employed, at least as far as the action of seizing the stone with gentleness, of reducing it to fragments, of taking these fragments and again reducing them, can be easy. But when the circumstances are no longer favourable, when the stone is large, the bladder irritable and contracted, his task is no longer the same; more skill, more tact are required; the instruments must be more powerful, more expeditious in their action, and the surgeon must also have the power of deciding quickly, what ought, and what ought not to be done.

The lithotritist cannot always immediately decide upon the propriety of operating or not; he is often obliged to hesitate: on one side circumstances induce him to believe that it is right to operate; others may lead him to reject the attempt, and he remains undecided. This indecision might be painful to him, did he not know that lithotrity presents the consolation of safely allowing attempts to be made. The more perfect are his instruments, the greater is their power on the stone; and the more gently he manœuvres them in the bladder, the greater is the confidence with which he may employ them; by being careful, and especially prudent, he may try, by applying the instrument itself, whether the operation will be advantageous to the patient." 352.

Here we must conclude. The last division of the work, containing a number of cases in which lithotrity was performed, will be noticed in our Periscope, and at convenient opportunities. We are informed by M. Heurteloup, that another volume is forthcoming, describing the manipulations of the lithotritist, and rendering the information on the subject as complete as he can give it. We need scarcely pronounce any formal criticism on the volume before us. Its perusal has given us a very high opinion of the ingenuity, talents, and judgment of the author. It will do him honour, and in all probability confer more substantial benefit. In parting we would offer one word of advice. Let the Baron be candid with the profession and the public, and he will ultimately find his account in it. Any of that coquetting quackery which inventors of particular arts, or professors of particular divisions of science, are too apt to indulge in, both lowers their respectability, and sooner or later affects their interests. We part from Baron Heurteloup with sentiments of esteem and respect.

